# Online Social Network Use by Health Care Providers in a High Traffic Patient Care Environment

**DOI:** 10.2196/jmir.2421

**Published:** 2013-05-17

**Authors:** Erik Black, Jennifer Light, Nicole Paradise Black, Lindsay Thompson

**Affiliations:** ^1^University of FloridaGainesville, FLUnited States

**Keywords:** social networking, emergency medicine, Internet

## Abstract

**Background:**

The majority of workers, regardless of age or occupational status, report engaging in personal Internet use in the workplace. There is little understanding of the impact that personal Internet use may have on patient care in acute clinical settings.

**Objective:**

The objective of this study was to investigate the volume of one form of personal Internet use—online social networking (Facebook)—generated by workstations in the emergency department (ED) in contrast to measures of clinical volume and severity.

**Methods:**

The research team analyzed anonymous network utilization records for 68 workstations located in the emergency medicine department within one academic medical center for 15 consecutive days (12/29/2009 to 1/12/2010). This data was compared to ED work index (EDWIN) data derived by the hospital information systems.

**Results:**

Health care workers spent an accumulated 4349 minutes (72.5 hours) browsing Facebook, staff cumulatively visited Facebook 9369 times and spent, on average, 12.0 minutes per hour browsing Facebook. There was a statistically significant difference in the time spent on Facebook according to time of day (19.8 minutes per hour versus 4.3 minutes per hour, *P*<.001). There was a significant, positive correlation between EDWIN scores and time spent on Facebook (*r*=.266, *P*<.001).

**Conclusions:**

Facebook use constituted a substantive percentage of staff time during the 15-day observation period. Facebook use increased with increased patient volume and severity within the ED.

## Introduction

There is an ever-increasing amount and diversity of online recreational opportunities available to Internet users, including online videogames, gambling, and social networking sites such as Facebook. Previous research has indicated that a majority of workers, regardless of age or occupational status, report engaging in personal Internet use in the workplace and misuse, such as this personal Internet use during work hours as well as more serious actions such as illegal downloading, accessing unauthorized information, and interpersonal aggression via email or other online communications, is increasingly prevalent [1-3]. Less is known about Internet use and misuse in medical settings and the potential impact on patient care.

Research by Morris-Docker et al showed that health professionals in patient care settings access the Internet during quiet periods throughout day and night for both work and non-work related activities without interfering with patient care or workload [4]. Since this study has been conducted, the Internet and the manner in which it is used has changed dramatically, particularly with the increased prevalence of social networking applications and websites and the prevalence of handheld access to the Internet. In this complex and rapidly developing technology landscape, online social networking applications have emerged as the significant Internet traffic destination. Previous research has established that health care professionals regularly use social networking applications to create and maintain relationships with friends, family, and coworkers [5-10]. While no known empirical study of social network use during clinical work hours has been conducted, studies of non-clinical workplace behavior and misbehavior have been a cornerstone of organizational behavior research [11].

Anecdotal evidence implied that Internet use, especially that associated with online social networking sites (such as Facebook), was common among individuals working in a level 4 trauma center and emergency department (ED) at an academic medical center in the Southeastern United States. Given Facebook’s increase in membership, coupled with the opportunity for access to the Internet on hospital workstations, this study aimed to investigate the volume of Facebook traffic generated by workstations in the ED in contrast to measures of its clinical volume and severity.

## Methods

### Sample Computers

The Institutional Review Board at the University of Florida approved this study. We analyzed network utilization records for 68 workstations located in the ED within one medical center for 15 consecutive days (12/29/2009 to 1/12/2010). These records were anonymous and no user information was available such as job description (physicians, resident, non-physician extender, nurse, clerical, or other staff) or personal demographics. The workstations were all Internet accessible and did not restrict access to popular social networking destinations (including Facebook), and all were located in open physician and staff workspaces within the ED. Workstations associated with designated break rooms were excluded from the study.

### Utilization and Volume/Severity Calculations

To calculate Facebook use, network utilization records for each workstation were obtained from the academic medical center’s SurfControl Web filtering software, which allows information technology administrators to categorize, track, and potentially limit user access to websites. SurfControl data yielded cumulative Facebook time from selected computers in one second increments. To control for instances in which an individual may have logged onto Facebook and then left the computer idle, SurfControl browse sensitivity was set at a maximum of 3 continuous minutes which roughly equates to the length of time a user is presumably actively engaged with a site. This conservatively takes into account “multi-tasking”, or being emergently needed in a different location, instead of counting the entire amount of time a webpage is open, which would likely over-estimate actual use. However, any true engagement with a webpage will only be counted as 3 minutes even if longer. To calculate how busy the ED was during the study, hourly ED work index (EDWIN) scores were calculated according to ED patient volume reports. An EDWIN score is a valid and reliable index that quantifies both the value and severity of ED admissions [12]. EDWIN is calculated using the following formula: n(i)t(i)/N(a)[B(T)-B(A)], where n(i) = the number of patients in the ED in triage category, t(i) = triage category, N(a) = number of attending physicians on duty, B(T) = number of treatment bays, and B(A) = number of admitted patients in the ED. The triage system recommended, the emergency severity index (ESI), was modified by reversing the ranking of triage categories; that is, an ESI score of 1 represented the least acute patient and 5 the most acute [13]. Data were analyzed using IBM SPSS PASW v.20, and included descriptive statistics and analysis of variance procedures with a *P*<.05 as considered significant.

## Results

In a 15-day period, health care workers spent an accumulated 4349 minutes (72.5 hours) browsing Facebook on workstations in one ED. ED staff cumulatively visited 9369 Facebook pages and spent, on average, 12.0 minutes per hour browsing Facebook.

**Figure 1:**
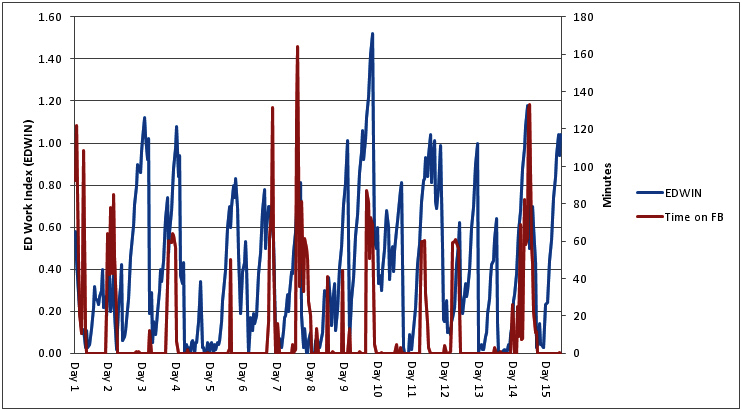
The relationship between Facebook use and ED business.

**Figure 2:**
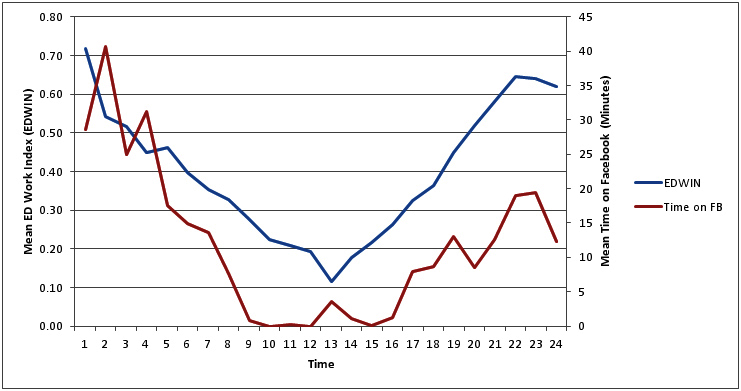
Average time on Facebook and average ED business across 24 hours.

There was a statistically significant difference in the amount of time spent on Facebook according to time of day. During the night shift, (7pm-7am), workers cumulatively spent an average of 19.8 minutes per hour browsing Facebook, yet during the day shift (7am-7pm) workers cumulatively spent an average of 4.3 minutes per hour browsing Facebook (*P*<.001). Of note, the ED was busiest during night shift hours (7pm-7am), with mean EDWIN ratings at 0.51 during night shifts versus 0.29 during day (*P*<.001). Importantly, there was a significant, positive correlation between EDWIN scores and time spent on Facebook (*r*=.266, *P*<.001), indicating that as the ED became busier, more time was spent browsing Facebook. (See Figure 1). The relationship between ED business and time on Facebook became more pronounced when averaged across the 15-day period (*r*=.757, *P*<.001, Figure 2).

## Discussion

### Principal Findings

Facebook use constituted a substantive percentage of staff time during the 15-day observation period, and Facebook use increased with increased patient volume and severity within the ED. It is understood that the near ubiquity of online entertainment, including social networking applications, can influence productivity, and, to the best of our knowledge, this study represented the first investigation of online social network use in an acute patient setting [2]. The results of the study provided evidence that social network use in the ED is common, which was expected, but also that it increased as patient volumes and acuity increased, which was unexpected. This unanticipated finding warrants further investigation since increased patient volume and acuity seemed to prompt health care workers to seek distractions or opportunities for cognitive time-outs, which is something that social networks provide. It is possible that these time-outs lead to improved worker functioning, but of more concern is that it may also represent a compromise of patient care. It could be argued that workers need to have the autonomy and authority to take breaks and relax while on the job. This notion is supported by research by Greenfield et al and Reinecke who found that engaging in brief tasks unrelated to work may have positive effects on worker fatigue and stress, yet promote increased worker productivity and happiness [14,15]. However, in specific care environments, such as EDs, individual, organizational, and environmental factors interact in a complex manner that could specifically impact patient health outcomes [16]. Future studies should examine if in acute environments, access to applications like Facebook should be relegated to break rooms or other designated spaces for non-work functions. While such a study would not and could not control access to online applications using mobile or cellular devices, which increasingly account for increasing amounts of traffic to social networking and other sites, it could send a message to health care workers that judicious use of accessing Internet sites is important for high quality care [17].

### Limitations

There are several limitations associated with this study that are worth noting. While the results indicated a significant amount of time was spent accessing Facebook, especially during times of increased patient acuity and volume, the study had no comparison to other non-Internet, non-clinical activities that staff may engage in during clinical shifts. Data collected from this study does not account for Internet use from personal mobile devices such as cell phones, where it is estimated that approximately 40% access the Internet or email [16]. Finally, Internet use of other Internet sites was not presented.

### Conclusions

Online social networking is an important and worthwhile activity to engage friends, family, and acquaintances, but as with other distractions, electronic or not, should be used cautiously in the workplace. It is our opinion that this level of Facebook use is unacceptably high in clinical spaces, and as such, computer workstations in patient-care space should limit access to online social networking and other forms of entertainment. Such limitations could be externally enforced through Web filters, such as SurfControl. However, internal regulation, such as encouraged by simple reminders like screensaver reminding staff about appropriate use during business hours, may better limit misuse. Given that many individuals now access online entertainment applications via mobile phones, the development and implementation of a comprehensive workplace Internet use policy, one that includes the use of personal mobile devices, should be considered.

## Multimedia Appendix 1

The relationship between Facebook use and ED business (with SD).

## Abbreviations

ED: emergency department
EDWIN: emergency department work index
ESI: emergency severity index
