# Supplementary figures and images for: Online Social Network Use by Health Care Providers in a High Traffic Patient Care Environment
Source: J Med Internet Res. 2013 May 17;15(5):e94. doi: 10.2196/jmir.2421 (PMC3668614; doi:10.2196/jmir.2421)

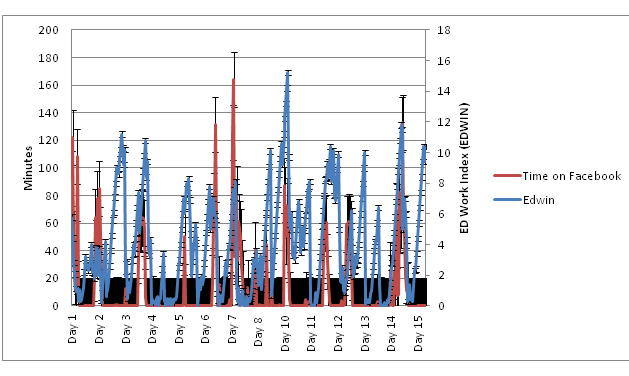

Supplement: Supplementary file 1 [file jmir_v15i5e94_app1.jpg]
